# Hyperhidrosis: Medical and Surgical Treatment

**Published:** 2008-04-18

**Authors:** Lewis P Stolman

**Affiliations:** Department of Dermatology, New York University School of Medicine, New York, NY

## Abstract

**Objective:** The treatment of hyperhidrosis, generalized or focal is a challenge for both physicians and surgeons. Focal hyperhidrosis—axillary, palmar, plantar, craniofacial—is the most common. Generalized hyperhidrosis is usually secondary to a systemic disorder or may be simply drug induced. Focal hyperhidrosis has its onset in childhood or adolescence and has a dramatic effect on one's quality of life. Medical, surgical, and electrical therapies can be employed to relieve hyperhidrosis in most patients. **Methods:** A review of the medical and surgical literature was performed to identify the usual causes and remedies for hyperhidrosis. **Results:** Specific treatments, medical and surgical are recommended for all affected individuals. **Conclusion:** Patients with hyperhidrosis need not suffer in silence. Etiologies can be identified for most. Safe and effective therapeutic options are available.

The primary function of the eccrine sweat glands is to assist in the maintenance of body temperature in response to heat exposure or exercise. *Hyperhidrosis* may be defined as sweating beyond what is necessary to maintain thermal equilibrium. It may be primary (idiopathic, essential) or secondary to a number of diseases and prescribed drugs. It may be localized, regionalized, or generalized.^1^ Regardless of the type or the cause of the hyperhidrosis, it is frequently socially embarrassing and occupationally disabling. Excess sweat on the hands may soil paper and artwork and make it virtually impossible to play many musical instruments. Careers in fields that require contact with paper, metal, and electrical components become unrealizable. Axillary and plantar hyperhidrosis may result in stains and cause damage to clothing and shoes. Generalized or regionalized hyperhidrosis may leave affected individuals with wet clothing that may have to be changed a number of times each day.

Physiologically, sweating is a function of the sympathetic nervous system. A sweat control center located in the preoptic area and anterior hypothalamus contains neurons that are sensitive to changes in internal temperature and also to cerebral cortical events. Sweat glands are innervated by sympathetic postganglionic fibers, but unlike ordinary sympathetic innervations the chemical mediator is acetylcholine. Sweating in response to thermal stimuli is generally acceptable and rarely a cause for complaint. Emotionally induced sweating tends to be localized to the palms, soles, and sometimes the forehead. Axillary sweating may be the result of both emotional and thermal stimuli.

The causes for generalized hyperhidrosis (Table 1) include a number of febrile illnesses, neoplastic and neurologic diseases, metabolic disorders, and drugs. The causes and conditions associated with localized hyperhidrosis (Table 2) include primary or focal hyperhidrosis, unilateral circumscribed hyperhidrosis, hyperhidrosis associated with intrathoracic neoplasms, olfactory hyperhidrosis, gustatory hyperhidrosis, spinal cord injuries, and Frey syndrome. Although primary or focal hyperhidrosis is the most common cause of palmoplantar hyperhidrosis, it may also occur in some patients with Raynaud's disease, rheumatoid arthritis, erythromelalgia, nail patella syndrome, keratosis palmaris et plantaris with clinodactyly, atrioventricular fistula, and cold injury. Whenever possible, the cause for hyperhidrosis should be identified and, if possible, treated.

*Primary* or *focal hyperhidrosis* is a disorder in which there is excess sweating of the hands, feet, face, and the axillae. A recent national survey estimated that 1.4% of the US population (4.0 million individuals) suffers from axillary hyperhidrosis.^2^ One third of these sufferers (1.3 million individuals) state that their sweating is barely tolerable or intolerable and frequently or always interferes with their daily life. Primary hyperhidrosis may be inherited and in contrast to generalized hyperhidrosis usually has its time of onset in childhood or adolescence.^3^ Palmar hyperhidrosis usually commences in childhood and axillary hyperhidrosis in adolescence. Characteristically, focal hyperhidrosis does not occur while sleeping. Primary hyperhidrosis is aggravated by heat and emotional stimuli. However, it is important to note that although emotional stimuli are necessary for primary hyperhidrosis to occur in affected individuals, it is not a psychological disease but rather a physiological disorder. It seems that in patients with primary hyperhidrosis the hypothalamic sweat centers are more sensitive to emotional stimuli of cerebral origin than in ordinary people. The occasional onset of primary hyperhidrosis in the neonatal period is evidence that this is far more than an emotional disorder!

A number of medical and surgical remedies are available for the treatment of hyperhidrosis. (Table 3)

## TOPICAL

Aluminum chloride and tanning agents are sometimes effective in the control of localized hyperhidrosis.^4^ Aluminum chloride may decrease sweating by mechanically obstructing eccrine sweat gland pores, although this mechanism has been disputed. The atrophy of the secretory cells seen in eccrine sweat glands exposed to aluminum chloride may account for the reduced sweating that most people enjoy with the use of aluminum chloride containing products. For most people with axillary hyperhidrosis, over-the-counter products are sufficient. For more severe sweating, aluminum chloride hexahydrate (20%) dissolved in anhydrous ethyl alcohol (Drysol) is frequently useful. The skin should be dry before application, because if moisture is present irritating hydrochloric acid forms. Washing just before application should be avoided. The optimum way to use the solution seems to be to apply it at bedtime to take advantage of the relative inactivity of sweat glands through the night and wash the product off first thing in the morning. Minor irritation can be relieved with the use of hydrocortisone creams. The solution should be used nightly until an effect occurs and then the interval between applications can be lengthened. Glutaraldehyde, tannic acid, and formaldehyde may be useful to treat palmar and plantar hyperhidrosis, but their tendency to stain the skin and in the case of formaldehyde solution its sensitizing potential limit their usefulness.^5^

## SYSTEMIC

For those patients whose hyperhidrosis is related to specific anxiety-producing events such as a speaking engagement, school dance, the use of a drug such as diazepam (Valium) may have an ameliorating effect. If hyperhidrosis is a part of a social anxiety disorder, fluoxetine (Prozac) may be a very useful therapeutic agent along with appropriate psychiatric care. Systemic anticholinergics may be helpful, but unfortunately the dosages required to achieve reduced sweating also result in adverse effects including xerostomia, mydriasis, cycloplegia, and bowel and bladder dysfunction. Most patients with localized or generalized hyperhidrosis cannot tolerate them for long. However, the anticholinergic oxybutynin (Ditropan) has been found to be useful in the relatively rare syndrome of episodic hyperhidrosis with hypothermia.^6^ Another anticholinergic, benztropine, was successfully used to treat hyperhidrosis in a patient with venlafaxine-induced excess sweating.^7^ Venlafaxine is an antidepressant that inhibits the reuptake of serotonin and norepinephrine. Sweating is said to occur in as many as 12% of all patients exposed to venlafaxine and other serotonin selective reuptake inhibitors. A nonsteroidal anti-inflammatory agent, indomethacin, in a dose of 25 mg tid was prescribed for a patient with arthritis who coincidentally had lifelong idiopathic generalized hyperhidrosis.^8^ Quite unexpectedly, she enjoyed a resolution of her lifelong hyperhidrosis. Although the mechanism for this beneficial effect is not clear, the information that prostaglandin E is found in increased amounts in the sweat of some patients with hyperhidrosis may offer an explanation.^9^ Influx of calcium from the extracellular to the intracellular space is necessary for the active secretion of sweat by eccrine sweat glands. This likely accounted for the improvement in palmar and plantar hyperhidrosis observed when a calcium channel blocker, diltiazem, was used to treat a family with palmar and plantar hyperhidrosis.^10^ Tricyclic antidepressants may cause excess sweating and yet, one would expect the anticholinergic action of tricyclics should block sweating not induce it. Clonidine (Catapres), a centrally active *α*-adrenergic autoreceptor stimulant, has been found to be useful in the treatment of hyperhidrosis because of tricyclics as well as menopause.^11^ Propoxyphene hydrochloride (Darvon), a narcotic and weak ganglionic blocking agent, may have an ameliorating effect on hyperhidrosis in patients with autonomic dysreflexia.^12^ *Autonomic dysreflexia* is a syndrome of sympathetic hyperactivity because of bladder or bowel distension, seen in some patients with spinal cord lesions at or above the 6th thoracic level (T6). Fludrocortisone acetate (0.3 mg daily) may control sweating in quadriplegics for whom orthostatic hypotension precipitates a sympathetic discharge.^13^ The reader is cautioned that many of these reports of therapeutic efficacy are anecdotal and all of these systemic agents carry with them the risk of side effects.

## IONTOPHORESIS

One of the simplest, safest, and most cost-effective treatments of palmar and/or plantar hyperhidrosis is that of *iontophoresis*, which is defined as the introduction of an ionized substance through intact skin by the application of a direct current. In 1936, Ichihashi used various solutions of atropine, histamine, and formaldehyde, and by iontophoresis demonstrated that sweating of the palms could be reduced.^14^ His work went relatively unnoticed until 1952 when Bouman and Gruenwald Lentzer published a report clearly demonstrating the efficacy of iontophoresis for the treatment of palmar and plantar hyperhidrosis in 113 patients.^15^ They demonstrated that the addition of an ionizable substance to the water was not necessary to obtain a therapeutic effect. Levit demonstrated a simple galvanic device that could be employed to relieve hyperhidrosis in 85% of affected patients.^16^^,^^17^

Although the exact mechanism of applying iontophoresis that relieves palmar or plantar hyperhidrosis is not known, it is thought to be due to poral plugging because the effect is reversed by cellophane tape stripping of the skin overlying eccrine sweat gland rendered euhidrotic by iontophoresis.^18^^,^^19^ For those patients who fail to respond to simple tap-water iontophoresis the addition of an anticholinergic directly to the tap water-filled treatment trays is frequently helpful. Although a number of devices are available for the administration of iontophoresis, this author prefers the Fischer MD-1a Galvanic unit and the technique employed is described in Tables 4 and 5. Side effects of iontophoresis are few. Occasionally the palms become too dry and may become cracked or fissured. This may be relieved by the use of moisturizers and/or a reduction in the frequency of treatments. Erythema and less frequently vesiculation of the skin may follow treatments and can be treated if necessary with simple 1% hydrocortisone cream. Compensatory hyperhidrosis does not occur. Iontophoresis is difficult to administer to the axillae and seems to cause more irritation than when administered to the palms and soles and thus is not as useful for the treatment of axillary hyperhidrosis.

## BOTULINUM TOXIN

Justinus Kerner, a German physician and poet, published the first comprehensive description of the symptoms of botulism between 1817 and 1822. He also proposed the possible therapeutic use of botulinum toxin, which he called *sausage poison*. Kerner's monograph describes the following illness from ingesting inadequately cooked smoked blood-sausages.

The tear fluid disappears, the gullet becomes a dead and motionless tube; in all mucous cavities of the human machine the secretion of the normal mucus stands still, from the biggest, the stomach, towards the tear canal and the excretory ducts of the lingual glands. No saliva is secreted. No drop of wetness is felt in the mouth, no tear is secreted any more—Urination can only be performed by standing and with difficulty. Extreme drying out of the palms, the soles, and the eyelids occurs.

Kerner went on to describe in humans and animals vomiting, intestinal spasms, mydriasis, ptosis, dysphagia, and in the end, respiratory failure. His reputation as an expert on sausage poisoning earned him the nickname *Sausage Kerner*. Subsequently sausage poisoning was named botulism from the Latin word for sausage, botulus. Years later in 1895 the bacterium responsible for botulism was identified by Emil-Pierre van Ermengem.^20^

Today, botulinum toxin is a useful therapeutic agent for the treatment of a number of diseases related to muscular dystonia. This potent toxin has proven to be a highly effective remedy for the treatment of conditions previously recalcitrant in the fields of ophthalmology, otorhinolaryngology, pediatrics, gastroenterology, and urology. The cosmetic denervation of muscles of facial expression using botulinum toxin has given dermatologists and plastic surgeons a new weapon against facial expression-induced wrinkles and lines. In recent years, certain types of hyperhidrosis have been successfully treated with botulinum toxin. Side effects reported following the local injection of botulinum toxin have been few and are usually related to undesired weakness in muscles adjacent to the treatment sites.

## TREATMENT OF AXILLARY HYPERHIDROSIS

Patients with quality-of-life impairing axillary hyperhidrosis, unresponsive to topical remedies, should be offered treatment with either botulinum toxin or surgical ablation of the axillary sweat glands. Because botulinum toxin inhibits the release of acetylcholine not only at neuromuscular junctions but also in postganglionic sympathetic fibers to sweat glands, it has been found to be useful to treat axillary hyperhidrosis.^21^^–^26 In the treatment of axillary hyperhidrosis as little as 50 units injected and distributed intradermally to each axilla can produce euhidrosis lasting as long as 6 months. With larger doses responses have been as long as 15 months. Many clinicians perform a starch-iodine test prior to the treatment to document the extent of the hyperhidrosis and to identify any so-called “hot spots,” which are areas that produce greater amounts of sweat. This author uses a simple facial tissue and a gentian violet marker to identify such areas. Generally, the hyperhidrosis occurs in the hair bearing part of the axillae and so it is in this area that the toxin should be placed. Discomfort is minimal and simple ice packs applied to the axillae prior to injection are sufficient for anesthesia. Botulinum toxin type A in an appropriate vehicle and applied directly to the axillae may prove to be an effective treatment.^27^

Patients with axillary hyperhidrosis who are unresponsive to topical therapy or do not want botulinum toxin or in whom it is contraindicated or who simply want a more long-lasting remedy may be offered a variety of surgical techniques designed to ablate the axillary sweat glands. The areas of greatest sweat production may be identified by draping a piece of facial tissue paper over the axilla or a starch-iodine test. Sometimes this area is quite small and a simple excision with closure is sufficient to remedy the problem.^28^ Patients in the past with moderate to severe hyperhidrosis have been offered more extensive procedures with undermining and resection of all exposed sweat glands.^29^ To obtain good closure and avoid limitation of movement because of cicatricial contracture, Z-plasty, and bat-shaped excisions and repairs can be employed.^30^ A promising arthroscopic shaver technique has recently been described, which is efficacious and is associated with a high-satisfaction rate and low morbidity.^31^ Alternative equally effective surgical treatments for axillary hyperhidrosis include subcutaneous liposuction with or without subcutaneous curettage.^32^^,^^33^

## TREATMENT OF PALMAR/PLANTAR HYPERHIDROSIS

Patients with palmar or plantar hyperhidrosis should be given a trial of a prescription strength antiperspirant such as aluminum chloride hexahydrate (20%) dissolved in anhydrous ethyl alcohol prior to botulinum toxin or iontophoresis treatments.^34^ Botulinum injections are very effective for the treatment of palmar hyperhidrosis.^35^^–^37 Generally the doses used are higher than those used for axillary hyperhidrosis ranging from 100 to 200 units of botulinum toxin type A per hand. Euhidrosis following such treatment may last for as long as a year.

Although effective, the clinical usefulness of this treatment is limited by the need for repetitive relatively painful injections, the cost of the botulinum toxin, and reports of weakness of the small muscles of the hands.^35^

Anesthesia is mandatory for the administration of botulinum toxin to the hand or feet. Although topical anesthesia in the form of lidocaine topical under occlusion for an hour followed by ice packs seems satisfactory for some, most practitioners have found the need for a either a Bier block or a block of the median, ulnar, and radial nerves.^38^ The Bier block, once learned, not only is safe and effective but also carries with it the advantage over nerve blocks of not affecting motor function. Thus patients can still use their hands immediately after the treatment. A simple and remarkably effective form of anesthesia is that of ice cube anesthesia during which an ice cube, held in gauze, is applied just prior to each individual injection of botulinum toxin type A.

Iontophoresis still remains a useful therapy because botulinum toxin injections for the treatment of palmar and plantar hyperhidrosis are not suitable for all patients.^34^ Specifically, musicians, surgeons, and others who depend on fine motor dexterity in their hands may not be able to accept the risks of diminished strength of their thumbs that may accompany botulinum toxin injections. Young children may not accept the discomfort involved in either regional nerve blocks or a Bier block for the anesthesia or even ice cube anesthesia for botulinum injections into the hands. Finally, iontophoresis is far less costly than botulinum treatments of palmar and plantar hyperhidrosis keeping in mind that from 100 to 200 units of botulinum toxin are used per hand and an anesthetic/administration fee may be involved.

## TREATMENT OF CRANIOFACIAL HYPERHIDROSIS

Craniofacial hyperhidrosis may respond to topical antiperspirants such as aluminum chloride hexahydrate (20%) dissolved in anhydrous ethyl alcohol or topical glycopyrrolate. Topical glycopyrrolate has been found to be useful to treat gustatory craniofacial hyperhidrosis in patients with diabetes.^39^ Botulinum toxin is very effective for craniofacial hyperhidrosis and is considered the treatment of choice for patients with Frey syndrome. *Frey syndrome* is characterized by unilateral gustatory hyperhidrosis that occurs in as many as 50% of patients who have undergone a parotidectomy.^40^ The most likely explanation is recurrence of parasympathetic nerve fibers that have lost their *target organ.* When botulinum toxin is used to treat craniofacial hyperhidrosis, the technique differs from that used to treat the facial musculature in that the injections are more superficial, that is, intradermal and evenly distributed over the area of hyperhidrosis. The area may be identified by either starch-iodine technique or a facial tissue held against the skin. Botulinum toxin, 50 to 100 units, should be administered at least 1½ cm above the supraorbital bridge to avoid a brow drop.

## SYMPATHECTOMY

Sympathectomy or upper thoracic (T2) ganglionectomy is frequently offered to patients with severe palmar hyperhidrosis and occasionally craniofacial hyperhidrosis. Lumbar sympathectomy is not usually employed for plantar hyperhidrosis because of the risk of sexual dysfunction. Although the efficacy of this procedure used in the treatment of palmar hyperhidrosis is not in doubt, with success rates of 92% to 99%, the complications are significant. Among the complications are compensatory hyperhidrosis (increased sweating in some other area of the body, 24% to 100%), gustatory sweating (sweating usually of the face related to the eating of foods), permanent Horner syndrome, wound infection, hemothorax, intercostal neuralgia, and recurrence of hyperhidrosis.^41^^–^50 The advent of endoscopic sympathectomy has reduced the incidence of many complications. Compensatory hyperhidrosis is the most common complication and the major reason for patient dissatisfaction with the procedure. Compensatory hyperhidrosis following sympathectomy can be far more life disrupting than palmar hyperhidrosis in that afflicted individuals may have to change sweat-soaked clothing 2 or 3 times a day. In a recent systematic literature review of sympathectomy for the treatment of hyperhidrosis, the question was asked, “Are we paying a high price for surgical sympathectomy?” One hundred thirty-five articles were reviewed reporting on 22,458 patients and 42,061 procedures, and 84.3% of the reported patients had the surgery for hyperhidrosis. Compensatory hyperhidrosis occurred in 52.3%, gustatory sweating in 32.3%, phantom sweating in 38.6%, and Horner syndrome 2.4%. In all, neuropathic complications occurred in 11.9% but were less common in patients who had the procedure for hyperhidrosis.^50^

Moran stated it quite succinctly,

“Complications related to the surgical approach, such as Horner's syndrome, brachial plexus injuries, pneumothorax and painful scars may occur, while following sympathectomy compensatory hyperhidrosis is usual and hyperhidrosis may recur.”^47^

## SUMMARY

The patient who complains of hyperhidrosis presents the physician with a diagnostic and therapeutic challenge. Patients who present with generalized hyperhidrosis are in general adults whose sweating occurs during both the waking and sleeping hours. Such patients require a search for a remedy that may sometimes be as simple as a drug that they are taking for some medical disorder. Occasionally a systemic illness may account for the onset of hyperhidrosis and a thorough examination and appropriate testing may be necessary to identify the cause. Most patients with primary or focal hyperhidrosis present in childhood or adolescence and have a problem localized to their axillae, hands, and/or feet. They have a neurocutaneous disorder not a psychiatric or endocrinologic disease. A number of systemic, topical, surgical, and electrical remedies are available for the treatment of hyperhidrosis. Patients with axillary hyperhidrosis may benefit from prescription-strength antiperspirants. For those who do not improve, botulinum toxin type A is frequently helpful. Surgical methods, refined in recent years, such as liposuction with or without curettage are effective alternative remedies. Patients with hyperhidrosis of the palm or soles, who fail to respond to topical agents, deserve a trial of conservative therapy, botulinum toxin, or iontophoresis, before aggressive surgical techniques that carry with them the risk of lifelong troublesome side effects are offered.

## Figures and Tables

**Table 1:** Hyperhidrosis

Generalized
Heat, humidity, and exercise
Febrile illnesses: acute and chronic infections
Neoplasia: Hodgkin's disease
Metabolic: carcinoid, diabetes mellitus, gout, hyperpituitarism, hypoglycemia, menopause, pheochromocytoma, and thyrotoxicosis
Sympathetic discharge: shock and syncope, intense pain, alcohol and drug withdrawal
Neurologic: Riley-Day syndrome, Ross syndrome, and irritative hypothalamic lesions
Drugs: propanolol, physostigmine, pilocarpine, tricyclic antidepressants, and venlafaxine

**Table 2:** Hyperhidrosis

Localized
Heat
Olfactory
Gustatory: citric acid, coffee, chocolate, peanut butter, and spicy foods
Neurologic lesions/Frey syndrome
Primary or focal hyperhidrosis

**Table 3:** Treatment of hyperhidrosis*

Medical
Topical
Antiperspirants: Aluminum hexahydrate in alcohol (Drysol)
2%–5% Tannic acid solution
5%–20% Formalin solution
10% Glutaraldehyde solution
Anticholinergics: 0.5% glycopyrrolate lotion
Systemic
Tranquilizers: diazepam (Valium), fluoxetine (Prozac)
Anticholinergics: propantheline bromide (Banthine/Probanthine), methoscopolamine bromide (Pamine), glycopyrrolate (Robinul), oxybutynin (Ditropan), & benztropine mesylate (Cogentin)
NSAIDs: indomethacin
Calcium channel blockers: diltiazem (Cardizem), Clonidine hydrochloride (Catapres), & botulinum toxin type A (Botox)
Surgical
Sympathectomy
Axillary sweat gland removal surgical procedures
Electrical
Iontophoresis

*NSAIDs indicates nonsteroidal anti-inflammatory drugs.

**Table 4:** Iontophoresis for hyperhidrosis with the Fischer Galvanic Unit*

A: Iontophoresis of hands using an assistant to control the unit	
1.	Fill the 2 plastic trays with tap water at room temperature to the top of the electrodes.
2.	With the Fischer unit off, connect the trays to the unit's outputs with the supplied cords.
3.	Make certain, the patient removes all jewelry and any small cuts or abrasions are covered with Vaseline or some similar water-resistant material.
4.	With the unit still off, have patient place 1 hand in each tray. The water level should be just above the skin of the tops of the fingers and hands. Remind the patient to keep hands in the water for complete duration. Removing the hands or touching the electrodes during the treatment may result in a slight shock. Because the intensity of the current flow is greatest at that part of the hand that is closest to the electrodes, instruct the patient to rotate their hands using a sliding motion away from the electrodes to avoid any unusual discomfort.
5.	Turn unit on with meter scale set from 0 to 50 and “intensity” knob at zero and gradually increase the amperage using the intensity knob to the therapeutic range of 15 to 18 mA, and treat for 10 min. (Note: If the red “active” light does not illuminate when you begin to increase current flow, return to zero and check all connections.)
6.	At the end of the 10 min, decrease current flow gradually to zero.
7.	When the meter indicates the flow is zero and the active light goes out, change the direction of current flow at the unit with the “Nor-Rev” toggle switch.
8.	Repeat steps (5) and (6) for 10 min. The total treatment duration will be 20 min.

*If the feet and not the hands are being treated, the patient once taught may adjust the controls him/herself.

**Table 5:** Iontophoresis for hyperhidrosis with the Fischer Galvanic Unit

B: Iontophoresis of the hands and feet without an assistant	
Note: This technique allows 1 hand to be free so that the patient can control the unit. However, the total duration of the treatment is increased from 20 to 40 min (20 min for each hand and foot combination).	
1.	Place 1 tray on a table and the other on the floor.
2.	Place 1 hand in 1 tray and a foot in the second.
3.	Follow instructions (5) to (8) above.
4.	Remove hand and foot and insert untreated hand and foot and repeat (5) to (8) above.
Notes	
1.	If the mineral content of the tap water is low and current flow is reduced, then the desired amperage (15 to 18 mA) may not be achieved. A teaspoonful of baking soda added and dissolved in each tray should remedy the problem.
2.	Patients need treatments every 2 to 3 days for 5 to 10 sessions before an effect is observed. Once euhidrosis is achieved, the interval between treatments may be stretched out. Some patients need only treat themselves once every 2 to 4 weeks.
3.	Avoid treating patients who are pregnant or have pacemakers.
4.	Patients who fail to respond to simple tap water iontophoresis may benefit from the addition of an anticholinergic to the water, ie, Robinul Forte 2 mg crushed and dissolved in each tray.
5.	Some patients experience irritation along the water line following treatment. One percent hydrocortisone cream is usually sufficient to relieve this.
